# Influence of interhospital transfer on endovascular thrombectomy outcome in acute ischemic stroke patients: an analysis of the TREAT-AIS registry

**DOI:** 10.3389/fneur.2026.1743928

**Published:** 2026-03-16

**Authors:** Ching-Yi Wang, Chi-Jen Chen, Yi-Chen Hsieh, Wey-Yil Lin, Sung-Chun Tang, Chih-Hao Chen, Chun-Jen Lin, Kuan-Hung Lin, Pi-Shan Sung, Chih-Wei Tang, Hai-Jui Chu, Chuan-Hsiu Fu, Chao-Liang Chou, Cheng-Yu Wei, Shang-Yih Yen, Po-Lin Chen, Hsu-Ling Yeh, Sheng-Feng Sung, Hon-Man Liu, Ching-Huang Lin, Meng Lee, I-Hui Lee, Yu-Wei Chen, Lung Chan, Li-Ming Lien, Hung-Yi Chiou, Jiunn-Tay Lee, Jiann-Shing Jeng

**Affiliations:** 1 Department of Neurology, Landseed International Hospital, Taoyuan City, Taiwan; 2 Department of Radiology, Taipei Hospital, Ministry of Health and Welfare, New Taipei City, Taiwan; 3 Ph.D. Program in Medical Neuroscience, College of Medical Science and Technology, Taipei Medical University, Taipei, Taiwan; 4 Department of Neurology, National Taiwan University Hospital, Taipei, Taiwan; 5 Department of Neurology, Neurological Institute, Taipei Veterans General Hospital, Taipei, Taiwan; 6 Department of Neurology, Chi Mei Medical Center, Tainan, Taiwan; 7 Department of Neurology, National Cheng Kung University Hospital, College of Medicine, National Cheng Kung University, Tainan, Taiwan; 8 Department of Neurology, Far Eastern Memorial Hospital, New Taipei City, Taiwan; 9 Department of Neurology, En Chu Kong Hospital, New Taipei City, Taiwan; 10 Department of Neurology, National Taiwan University Hospital Hsin-Chu Branch, Hsinchu, Taiwan; 11 Department of Neurology, Mackay Memorial Hospital, Taipei, Taiwan; 12 Department of Neurology, Chang Bing Show Chwan Memorial Hospital, Changhwa City, Taiwan; 13 Department of Neurology, Tri-Service General Hospital, National Defense Medical Center, Taipei, Taiwan; 14 Department of Neurology, Taichung Veterans General Hospital, Taichung, Taiwan; 15 Department of Neurology, Shin Kong WHS Memorial Hospital, Taipei, Taiwan; 16 Division of Neurology, Department of Internal Medicine, Ditmanson Medical Foundation Chia-Yi Christian Hospital, Chiayi City, Taiwan; 17 Department of Medical Imaging, Fu Jen Catholic University Hospital, New Taipei City, Taiwan; 18 Department of Neurology, Kaohsiung Veterans General Hospital, Kaohsiung, Taiwan; 19 Department of Neurology, Chang Gung University College of Medicine, Chang Gung Memorial Hospital Chiayi Branch, Puzi City, Chiayi County, Taiwan; 20 Department of Life Sciences, National Central University, Taoyuan, Taiwan; 21 Department of Neurology, School of Medicine, College of Medicine, Taipei Medical University, Taipei, Taiwan; 22 Department of Neurology, Taipei Medical University-Shuang Ho Hospital, New Taipei City, Taiwan; 23 School of Public Health, College of Public Health, Taipei Medical University, Taipei, Taiwan

**Keywords:** direct transport, endovascular thrombectomy, functional outcome, inter-hospital transfer, ischemic stroke, onset-to-puncture time

## Abstract

**Background:**

The outcome of patients undergoing endovascular thrombectomy (EVT) for large vessel occlusion in a comprehensive stroke center (CSC) is affected by the onset-to-treatment time. Whether the pathway to CSC arrival (direct vs. interhospital transfer) is thus associated with EVT outcomes among such patients is unknown.

**Methods:**

Using the Taiwan Registry of Endovascular Thrombectomy for AIS registry, patients ≥20 years of age and receiving EVT for AIS within 24 h of onset between January 2019 and December 2022 were included. Patients were categorized according to CSC arrival pathway into direct arrival and transfer groups. The primary outcome was 3-month functional independence, defined as a modified Rankin Scale (mRS) of 0–2.

**Results:**

Of the1830 patients included, 79% arrived at a CSC directly and 21% via transfer. More patients in the direct arrival than the transfer group achieved a 3-month mRS of 0–2. A significant interaction was found between onset-to-puncture (OTP) time and arrival pathway for achieving 3-month mRS 0–2 (*p*_*interaction*_ = 0.017). Arrival by transfer was associated with reduced odds of achieving mRS 0–2 when OTP time was < 6 h (aOR, 0.55), but with increased odds when OTP was ≥6 h (aOR, 1.95).

**Conclusions:**

Direct arrival was associated with improved outcomes if OTP < 6 h. Patients who arrive via transfer may still benefit substantially from EVT during the later treatment window. Further study is warranted to examine the predictors of favorable post-EVT outcomes within a 24-h window to facilitate timely treatment.

## Introduction

Endovascular thrombectomy (EVT) is the guideline-recommended standard-of-care for patients with acute ischemic stroke (AIS) caused by large vessel occlusion (LVO) (1, 2). The effectiveness of EVT for improving patients' functional outcomes is tightly linked to the time until treatment, with studies showing that each hour of delay in receiving EVT decreases the probability of achieving functional independence by 5.3% (3). For each hour of delay in receiving thrombectomy after arrival at a EVT-capable facility, an estimated 0.92 healthy life-years are lost (4). EVT requires specialized infrastructure and medical expertise that are often available only at comprehensive stroke centers (CSC), which may be less accessible than local primary stroke centers (PSC) that provide intravenous thrombolysis (IVT) alone. The transport strategy for patients with suspected LVO requires careful considerations and appropriate triage between healthcare services to avoid overcrowding and misdistribution of medical resources, delayed treatment decisions, and poorer recovery.

Previous observational studies and *post-hoc* analyses comparing post-EVT clinical outcomes between LVO patients according to transport strategy mostly supported direct transport to a CSC for EVT to achieve better 3-month functional independence. A meta-analysis of multiple studies found that patients admitted via direct transport to a CSC for mechanical thrombectomy were 1.3 times more likely to achieve better functional outcomes than were secondary-transfer patients, despite comparable recanalization rates (5). Similarly, Romoli et al. reported that, compared to patients who underwent IVT at a PSC before transfer to a CSC (‘drip-and-ship' approach), those who were directly transported to a CSC (mothership approach) had better 3-month functional independence. Additionally, delayed administration of thrombolysis was associated with poorer functional outcomes. Nevertheless, two multi-center randomized controlled trials reported less conclusive findings. The RACECAT trial studied a cohort of patients with suspected LVO stroke who were randomly assigned to an EVT-capable CSC (482 patients) or a local PSC (467 patients) for treatment (6). Despite significantly higher EVT and lower IVT rates in those directly transported to a CSC, no statistically significant differences in post-treatment outcomes or mortality were observed at 90 days between the two types of treatment centers (7). Comparably, the TRIAGE-STROKE study reported a statistically non-significant increase in the odds of functional improvement at 90 days for patients who were transported to a CSC first among the 104 enrolled patients with AIS (8). However, the small sample size could have resulted a power too low to show a significant benefit in the direct-to-CSC approach (8).

Notably, the location and distribution of stroke centers, population density, and healthcare structure vary significantly across geographical regions. These factors could affect the efficiency of triage and transport and whether or not AIS patients were able to receive timely treatment (9, 10). These factors indicate the need to conduct country- or region-specific studies regarding the treatment center type and patients' functional outcomes following an AIS diagnosis. Studies from East Asia are more scarce than those from Western countries. Few studies have been conducted in Taiwan, where the population density is high and nearly all residents are covered by National Health Insurance (11). Previous studies highlight the importance of considering onset-to-puncture (OTP) time, defined as the duration of time between the onset of symptoms and the administration of corresponding treatments, which is clearly delayed in transferred patients. Thus, this study aims to examine the association between patient functional outcomes and CSC arrival status, clinical factors other than OTP time, and neuroimaging factors using data in the nation-wide Taiwan Registry of Endovascular Thrombectomy for Acute Ischemic Stroke (TREAT-AIS).

## Methods

### Study participants

We conducted retrospective analysis of data in the TREAT-AIS, a nation-wide prospective, multicentered, observational registry of Taiwanese residents (*n* = 1830). Details of the registry design and included data have been published previously (12). Briefly, this registry is a joint effort involving 10 medical centers and 9 teaching hospitals started in January 2019, following approval by the institutional review boards of all participating hospitals (ClincalTrials.gov number: NCT05281055). Patients who met the following criteria were included in the registry: (1) age ≥ 20 years (the threshold was set according to the Civil Code in effect prior to 2023, which defined adulthood as beginning at 20 years; thus, cases enrolled before 2023 followed this legal definition); (2) received EVT for AIS caused by intracranial LVO within 24 h of stroke onset; (3) presented with LVO confirmed by magnetic resonance imaging angiography, computed tomography angiography, or digital subtraction angiography; (4) had pre-stroke independence defined by a modified Rankin Scale (mRS) score of 0–2; and (5) consented to participate. For the purpose of this study, patients who underwent EVT for AIS from January 2019 to December 2022 were included.

The study was conducted in accordance with the principles and guidelines outlined in the Declaration of Helsinki. TREAT-AIS received approval from the joint institutional review board of all participating hospitals. This study was approved by the Institutional Review Board of Landseed International Hospital (LIHIRB-20-027B1). Informed consent was waived for this observational cohort study by the Institutional Review Board of Landseed International Hospital.

### Data categorization and collection

All participating hospitals were EVT-capable CSCs. Patients were categorized into two groups depending on their pathway to arrival at the CSC: either (1) arriving directly at the CSC (‘direct' group) or (2) via transfer from a PSC (‘transfer' group). Eligibility for EVT was determined based on the recommendations by the American Heart Association or the Taiwan Stroke Society (1, 2). Participant demographics and clinical data were collected at three workflow timepoints: symptom onset, pathway to CSC arrival, and EVT initiation (‘puncture'). The following clinical data were included in the analysis: pre-admission data, risk factors, medication history, baseline stroke severity according to the National Institutes of Health Stroke Scale (NIHSS), Alberta Stroke Program Early CT Score (ASPECTS; in the transfer group, the score was determined at the CSC based on imaging performed at the PSC. New imaging and ASPECTS reassessment were conducted upon CSC arrival only when there was a significant temporal delay between the PSC scan and CSC arrival, or when patients exhibited clear clinical deterioration during transfer), Glasgow Coma Scale (GCS), pharmacological and/or mechanical treatments received before EVT at the CSC or PSC, and medications given at the time of discharge. The efficacy and safety outcomes of stroke-related treatments were adjudicated by the principal investigators at each site.

### Treatment and safety outcomes

The primary treatment outcome was functional independence (mRS range, 0–2) at 3 months post-EVT. The secondary outcomes were as follows: (1) successful post-EVT recanalization (defined as modified Thrombolysis in Cerebral Infarction [mTICI] grades 2b−3); (2) mRS at 3 months post-EVT; and (3) mortality. The safety outcome was the incidence of symptomatic intracranial hemorrhage (sICH), defined as type 2 parenchymal hemorrhage with ≥4-point deterioration according to the NIHSS evaluated within 36 h after baseline.

### Statistical analysis

Continuous variables were analyzed using Student's *t*-test. Categorical variables were analyzed using the Chi-squared test or Fisher exact test and are presented as frequencies and percentages. Multivariate logistic regression analysis was performed to assess EVT outcomes and clinical factors. The effect of OTP time on outcomes according to the arrival pathway was examined. A p _interaction_ was derived from the interaction term (OTP × arrival group) in adjusted models. A *p*-value < 0.05 was considered statistically significant. All statistical analyses were performed using the SAS software (version 9.4, SAS Institute, Cary, NC).

## Results

### Patient characteristics

Between January 2019 and December 2022, a total of 1830 patients registered in the TREAT-AIS received EVT for AIS in a participating CSC. Of these patients, 1448 patients (79.1%) arrived directly at the CSC, and 382 (20.9%) were transferred from a PSC. Patient demographics and baseline clinical data are shown in Table 1. The mean age of the two groups was comparable (71.2 and 71.1 years for the direct and the transfer groups, respectively). Compared to the transfer group, the direct group had a higher rate of previous cerebrovascular accident, dyslipidemia, and cancer. The direct group also had more patients using antiplatelet and lipid-lowering medications before admission, with higher median GCS and ASPECTS scores than the transfer group. However, patients in the direct group had less carotid occlusion and lower NIHSS at admission than did those in the transfer group (all *p* < 0.05). All other characteristics, including the fraction of patients treated with IVT before EVT, and timing of presentation were comparable between arrival pathways (Table 1).

**Table 1:** Demographics of patients who arrived at a CSC for EVT.

**Variable**	Direct arrival (***N*** = 1,448)		Transferred from PSC (***N*** = 382)		***P***
Age, mean and SD	71.2	13.6	71.1	12.8	0.949
Sex, n and % female	–	–	–	–	–
	627	43.3	173	45.3	0.486
**Risk factors**, ***n*** **and %**					
Hypertension	1,049	72.4	282	73.8	0.591
Diabetes mellitus	493	34.0	140	36.6	0.342
Previous cerebrovascular accident	309	21.3	64	16.8	**0.048**
Previous transient ischemic attack	37	2.6	6	1.6	0.259
Dyslipidemia	767	53.0	174	45.5	**0.010**
Cancer	203	14.0	35	9.2	**0.012**
Smoking	408	28.2	110	28.8	0.811
**Heart disease**, ***n*** **and %**					
Atrial fibrillation	750	51.8	212	55.5	0.197
Other ischemic conditions (CAD, old MI)	212	14.6	51	13.4	0.523
**Medication before admission**, ***n*** **and %**					
Anticoagulant	228	15.7	50	13.1	0.198
Antiplatelet	286	19.8	57	14.9	**0.031**
Lipid-lowering drug	277	19.1	50	13.1	**0.006**
GCS score, median and IQR	11	9–15	11	9–14	**0.025**
NIHSS at admission, median and IQR	17	12–23	19	14–24	**0.004**
ASPECTS, median and IQR (*n* = 1,786)	9	7–10	8	6–9	**< 0.001**
**Target vessel**, ***n*** **and %**					
Carotid	350	24.2	120	31.4	**0.004**
M1	839	57.9	234	61.3	0.242
M2–3	341	23.5	58	15.2	**< 0.001**
Anterior cerebral artery	41	2.8	6	1.6	0.166
Posterior, including VA, BA, and PCA	156	10.8	48	12.6	0.322
Intravenous thrombolysis, n and % (*n* = 1,736)	536	39.0	131	36.4	0.373
**Time of day**, ***n*** **and %**					
Day (08:00–17:00)	851	58.8	201	52.6	0.053
Night	582	40.2	179	46.9	
**Weekend**, ***n*** **and %**					
No	1,055	72.9	289	75.7	0.273
Yes	378	26.1	92	24.1	
**COVID-19 pandemic**, ***n*** **and %**					
No (2019–2020)	580	40.1	149	39.0	0.342
Yes (2021–2022)	853	58.9	232	60.7	

*P* < 0.05 shown in bold.

ASPECTS, Alberta Stroke Program Early Computed Tomography Score; BA, basilar artery; CAD, coronary artery disease; CSC, comprehensive stroke center; EVT, endovascular thrombectomy; GCS, Glasgow Coma Scale; IQR, interquartile range; MI, myocardial infarction; NIHSS, National Institute of Health Stroke Scale; PCA, posterior cerebral artery; PSC, primary stroke center; VA, vertebral artery.

### Post-EVT outcomes

**Figure 1:**
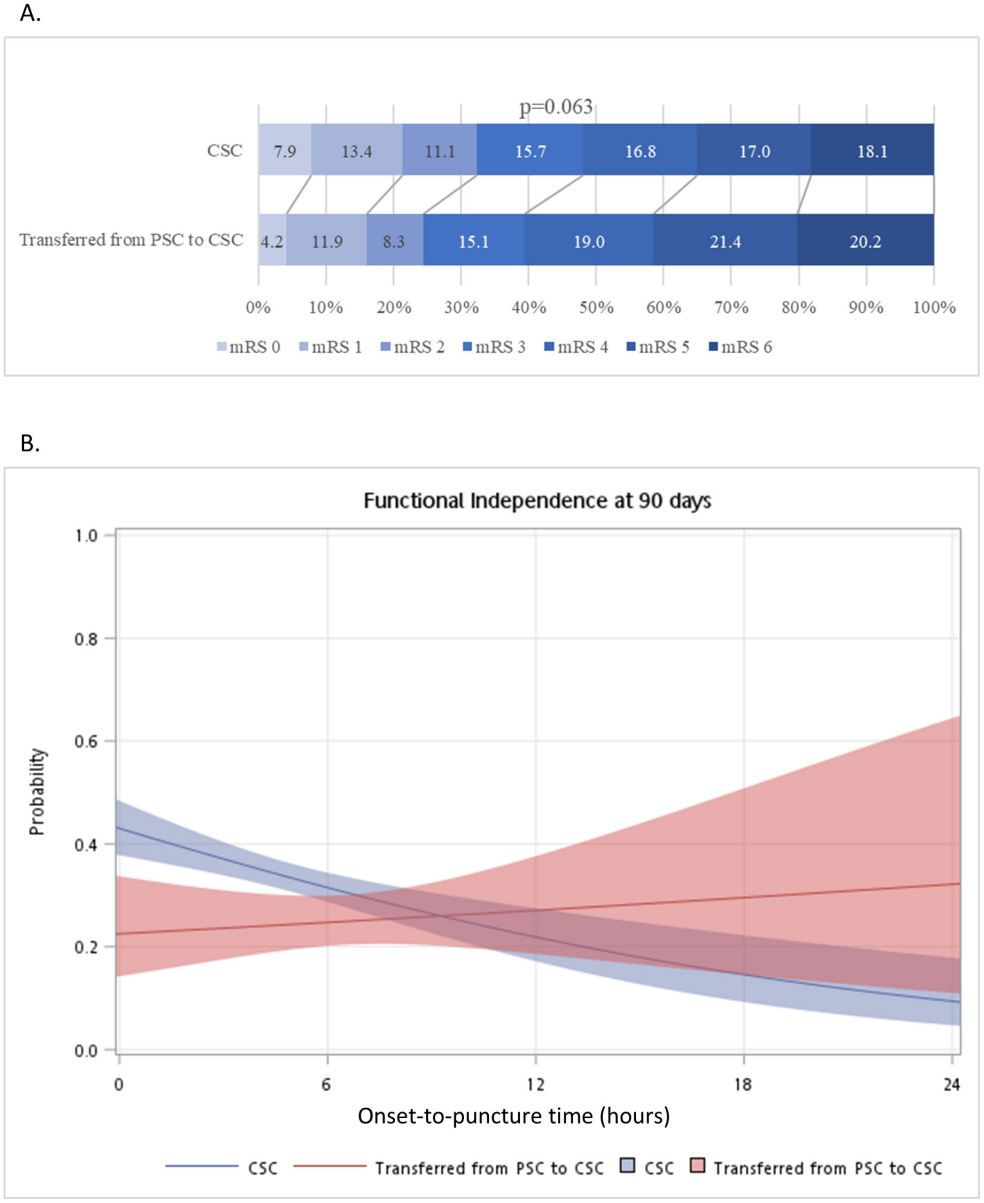
**(A)** Distribution of 3-month modified Rankin Scale (mRS) score by arrival pathway. **(B)** Interaction between onset-to-puncture time and arrival pathway on 3-month functional independence (mRS 0–2) outcome. The effect of onset-to-puncture time on the probability of achieving 3-month functional independence was compared between patients who arrived at a CSC directly (blue line) and those who were transferred from a PSC (red line). *P* = 0.017 for interaction between arrival pathways.

The direct and transfer groups did not differ significantly (p > 0.05) with respect to the rate of successful recanalization (defined as mTICI grades 2b−3; 82.9% and 86.8%, respectively), sICH (4.4% and 7.9%), and 3-month mortality (16.7% and 18.9%) (Table 2). The distribution of mRS scores at 3 months after EVT did not differ significantly between the two groups (*p* > 0.05) (Figure 1A). However, the proportion of patients achieving 3-month functional independence (defined as mRS 0–2) was significantly higher in the direct group than in the transfer group (30.5 and 24.3%, respectively; *p* < 0.001) (Table 2). Timing of presentation did not significantly affect post-EVT outcomes (Supplementary Tables S1–S6), except that among direct arrivals, weekday presentations were associated with higher 3-month mRS mortality than weekend presentations (18.0 and 12.9%, respectively; *p* = 0.024) (Supplementary Table S3).

**Table 2:** Comparison of EVT outcomes between direct CSC arrival and transfer from a PSC.

**Outcome**	**Direct arrival**	**Transfer from a PSC**	***P***
Total Cases	*N* = 1,448	*N* = 382	–
**Functional outcomes**, ***n*** **(%)**			
Final TICI (mTICI 2b−3)	1,201 (82.9)	331 (86.8)	0.081
Symptomatic ICH	63 (4.4)	30 (7.9)	0.898
3-month mRS 0–2 (*n* = 1,661)	429 (30.5)	82 (24.3)	**0.004**
3-month mRS mortality	242 (16.7)	72 (18.9)	0.325
**Workflow outcomes, median (IQR) (min)**			
Onset-to-door (*n* = 1,477)	73 (40–170)	236 (180–324)	**< 0.001**
Difference vs. transfer, (min)	163	–	–
Door-to-puncture (*n* = 1,626)	151 (116–193)	103 (74.5–131.5)	**< 0.001**
Difference vs. transfer, (min)	−48	–	–
Onset-to-puncture (*n* = 1,611)	229.5 (174–345)	336 (274–430)	**< 0.001**
Difference vs. transfer, (min)	106.5	–	–
Onset-to-puncture < 6 h^*^, *n* (%) (*n* = 1,611)	974 (76.8)	199 (58.0)	**< 0.001**
**Early treatment window (onset-to-puncture time**<**6 h)**			
**(*****n*** = **1,173)**			
3-month mRS 0–2, *n* (%) (*n* = 1,060)	319 (36.0)	39 (22.3)	**< 0.001**
Symptomatic ICH, *n* (%) (*n* = 1,173)	43 (4.4)	17 (8.5)	**0.016**
Door-to-puncture, median (IQR)(min) (*n* = 1,063)	140 (109–173)	91.5 (68–114)	**< 0.001**
**Late treatment window (onset-to-puncture time** ≥**6 h)**			
**(*****n*** = **438)**			
3-month mRS 0–2 (%) (*n* = 409)	69 (24.7)	37 (28.5)	0.423
Symptomatic ICH (%) (*n* = 438)	9 (3.1)	8 (5.6)	0.204
Door-to-puncture, median (IQR)(min) (*n* = 305)	191 (144–303)	118.5 (86–162)	**< 0.001**

*P* < 0.05 shown in bold.

^*^Time (< 6 vs. >6 h) × arrival pathway (PSC vs. CSC) for mRS 0–2; *P* interaction = 0.005.

CSC, comprehensive stroke center; door, arrival at CSC; EVT, endovascular thrombectomy; ICH, intracranial hemorrhage; IQR, interquartile range; mRS, modified Rankin Scale; mTICI, modified Thrombolysis in Cerebral Infarction; Onset, symptom onset; PSC, primary stroke center; puncture, EVT initiation; TICI, Thrombolysis in Cerebral Infarction.

### Workflow outcomes

The direct group had a significantly shorter onset-to-door time (73.0 min vs. 236.0 min) and onset-to-puncture time (229.5 min vs. 336.0 min) than did the transfer group. Notably, when door-to-puncture time was uniformly defined as the interval from arrival at the CSC to arterial puncture, the direct group had a longer door-to-puncture time (151.0 min) than the transfer group (103.0 min; all *p* < 0.001), reflecting that some diagnostic steps may have been completed before CSC arrival in transferred patients. The proportion of patients who underwent EVT within 6 h was significantly higher in the direct group (76.8%) than in the transfer group (58.0%; *p* < 0.001) (Table 2).

Patients presenting directly to the CSC during nighttime hours experienced longer onset-to-puncture times compared to daytime arrivals (236.5 min vs. 225 min; *p* = 0.049) (Supplementary Table S1). No significant diurnal variations in workflow metrics were observed among patients transferred from a PSC (Supplementary Table S2). Direct arrivals on weekends had longer door-to-puncture times than weekday arrivals (158 min vs. 147 min; *p* = 0.007) (Supplementary Table S3). Similarly, PSC-to-CSC transfers on weekends showed longer door-to-puncture times than weekday transfers (111.5 min vs. 98.5 min; *p* = 0.039), although onset-to-door time was shorter on weekends (224.5 min vs. 240 min; *p* = 0.049) (Supplementary Table S4). Workflow outcomes remained consistent regardless of the COVID-19 pandemic for both cohorts (Supplementary Tables S5, S6).

Among all patients with logged PSC arrival times (*n* = 99), the median time from onset to PSC arrival was 60 min (interquartile range [IQR] 37–100), while the median time from onset to CSC arrival was 223 min (IQR 178–285), a difference of 163 min (*p* < 0.001). The median time from PSC arrival to puncture was 233 min (202–273), compared with 82 (63–101) min from CSC arrival to puncture, yielding a difference of 151 min (*p* < 0.001).

### Post-stroke outcomes

Factors potentially associated with 3-month functional independence are presented in Supplementary Table S7. Compared to patients who arrived directly, those who were transferred were less likely to achieve 3-month functional independence (univariable logistic regression analysis: OR, 0.67; *p* = 0.004). Univariable analysis identified age, hypertension, cancer, atrial fibrillation, baseline NIHSS score, carotid artery involvement, and OTP time >6 h as risk factors for failing to achieve 3-month functional independence (all *p* < 0.05). Undergoing IVT before EVT and higher ASPECTS and GCS scores were positively associated with 3-month functional independence (all *p* < 0.05). After adjustment for the risk factors identified by multivariate analysis, age (aOR, 0.96), history of cancer (aOR, 0.50), NIHSS score at baseline (aOR, 0.94), IVT (aOR, 1.41), ASPECTS score (aOR, 1.14), and GCS score (aOR, 1.07) were significant factors associated with 3-month functional independence (Supplementary Table S7).

### Role of OTP time

The role of OTP time and its potential interaction with arrival pathway was tested by stratification and logistic regression. Among patients treated earlier (OTP < 6 h), the direct group had higher proportion of patients achieving functional independence than the transfer group (36.0 vs. 22.3%) and fewer with sICH (4.4 vs. 8.5%,), despite a longer door-to-puncture time (140.0 min vs. 91.5 min; all *p* < 0.05) (Table 2). However, the differences in functional independence and sICH between the two arrival pathways were statistically insignificant for patients treated later (OTP, 6–24 h).

Figure 1B shows the relationship between time to treatment and functional independence for both arrival pathway groups. The rate of functional independence decreased exponentially in the direct group and intersected with the transfer group at approximately 8–10 h after onset, leading to a significant interaction (*p*_*interaction*_ = 0.017).

Supplementary Tables S8, S9 present descriptive distributions and univariate analyses for each variable associated with 3-month functional independence and sICH within the < 6-h and ≥6-h strata. Adjusted multivariable results for factors potentially associated with these outcomes in the early and late treatment windows are presented in Table 3. In the early treatment window, patients transferred from a PSC were less likely to exhibit 3-month functional independence (aOR, 0.55; *p* = 0.006), compared to those who arrived directly at a CSC. In addition to the arrival pathway, multivariate analysis identified age (aOR, 0.97), NIHSS at baseline (aOR, 0.94), IVT (aOR, 1.53), ASPECTS score (aOR, 1.10), and GCS score (aOR, 1.07) as factors significantly associated with 3-month mRS of 0–2 (all *p* < 0.05) (Table 3). Among patients treated in the early time window, the pathway of arrival at a CSC was not associated with developing sICH or with factors relevant to the development of sICH, including dyslipidemia (aOR, 0.52), lipid-lowering drug use (aOR, 2.18) and ASPECTS score (aOR, 0.86; all *p* < 0.05) (Table 3).

**Table 3:** Multivariate logistic regression analysis of 3-month mRS 0–2 and sICH between subjects with early and late treatment.

**Variable**	Onset-to-puncture time < 6 h				Onset-to-puncture time ≥ 6 h			
	3-month mRS 0–2		Symptomatic ICH (%)		3-month mRS 0–2		Symptomatic ICH (%)	
	**aOR (95% CI)**	***P***	**aOR (95% CI)**	***P***	**aOR (95% CI)**	***P***	**aOR (95% CI)**	***P***
Transferred from PSC (Ref: Direct arrival)	0.55 (0.36–0.84)	**0.006**	1.84 (0.99–3.42)	0.055	1.95 (1.05–3.62)	**0.033**	1.58 (0.53–4.75)	0.417
Age	0.97 (0.96–0.98)	**< 0.001**	1.00 (0.98–1.02)	0.914	0.95 (0.93–0.97)	**< 0.001**	1.00 (0.96–1.05)	0.940
Hypertension	0.87 (0.62–1.21)	0.400	1.83 (0.85–3.93)	0.120	0.78 (0.42–1.46)	0.441	1.69 (0.44–6.55)	0.446
Previous transient ischemic attack	2.40 (0.86–6.66)	0.093	N/A	N/A	1.56 (0.35–6.99)	0.561	N/A	N/A
Dyslipidemia	0.96 (0.70–1.31)	0.795	0.52 (0.28–0.97)	**0.040**	0.94 (0.53–1.65)	0.818	0.78 (0.26–2.35)	0.981
Cancer	0.48 (0.30–0.76)	**0.002**	0.25 (0.06–1.06)	0.060	0.51 (0.22–1.17)	0.113	2.59 (0.70–9.56)	0.659
Atrial fibrillation	1.02 (0.75–1.39)	0.907	0.84 (0.47–1.50)	0.566	1.42 (0.79–2.54)	0.242	1.35 (0.42–4.36)	0.154
Antiplatelet medication	0.98 (0.67–1.42)	0.915	0.95 (0.48–1.90)	0.891	0.37 (0.16–0.87)	**0.023**	1.37 (0.37–5.04)	0.622
Lipid-lowering drug	1.36 (0.91–2.03)	0.133	2.18 (1.05–4.54)	**0.038**	1.85 (0.82–4.16)	0.138	0.74 (0.14–4.01)	0.730
NIHSS at baseline	0.94 (0.92–0.97)	**< 0.001**	1.03 (0.98–1.08)	0.313	0.91 (0.87–0.95)	**< 0.001**	1.02 (0.94–1.12)	0.634
Intravenous thrombolysis	1.53 (1.14–2.05)	**0.005**	1.20 (0.69–2.10)	0.515	0.73 (0.34–1.55)	0.411	0.39 (0.05–3.30)	0.386
ASPECTS before EVT	1.10 (1.02–1.20)	**0.021**	0.86 (0.76–0.98)	**0.023**	1.29 (1.09–1.52)	**0.003**	0.99 (0.76–1.29)	0.950
Carotid artery involved (Ref: No)	0.92 (0.65–1.28)	0.612	1.30 (0.71–2.36)	0.398	0.56 (0.27–1.13)	0.104	2.48 (0.85–7.23)	0.097
M2–3 artery involved (Ref: others)	1.08 (0.76–1.53)	0.675	0.87 (0.43–1.77)	0.709	1.66 (0.86–3.22)	0.132	N/A	N/A
GCS score	1.07 (1.02–1.14)	**0.012**	1.05 (0.95–1.16)	0.364	1.06 (0.98–1.15)	0.171	1.01 (0.85–1.22)	0.877

*P* < 0.05 shown in bold.

aOR, adjusted odds ratio; ASPECTS, Alberta Stroke Program Early Computed Tomography Score; CI, confidence interval; CSC, comprehensive stroke center; EVT, endovascular thrombectomy; GCS, Glasgow Coma Scale; ICH, intracranial hemorrhage; mRS, modified Rankin Scale; NIHSS, National Institute of Health Stroke Scale; PSC, primary stroke center; OR, odds ratio.

Nevertheless, multivariate logistic regression analysis showed that the arrival pathway was positively associated with 3-month functional independence (aOR, 1.95; *p* = 0.033) among patients in the later time window (Table 3). Other significant factors included age (aOR, 0.97), antiplatelet medication use (aOR, 0.37), NIHSS at baseline (aOR, 0.91), and ASPECTS score (aOR, 1.29); (all *p* < 0.05). No factors were found to be associated with sICH among patients treated during the later time window (*p* > 0.05).

## Discussion

In this study, we found that the arrival pathway significantly influenced the 3-month functional independence of patients who received EVT for LVO. However, the arrival pathway did not affect the rate of successful recanalization, sICH, or 3-month mortality. Significant differences in arrival and treatment times were found between the two arrival pathways. The transfer group had shorter door-to-puncture time than did the direct group, presumably due to earlier IVT, (7) supported by a comparable percentage of patients who underwent IVT before EVT in both groups, imaging evaluation in a PSC, and surgical preparation in advance. However, patients who were transferred from a PSC might be negatively affected by longer door-in–door-out and traffic times than those who arrived directly. Consequently, the proportion of patients receiving timely EVT (i.e., OTP < 6 h) was lower among the patients who were transferred. Multivariate analysis demonstrated a significant association between OTP time and 3-month functional independence, highlighting the interaction between timely treatment and arrival pathway on 3-month functional outcomes.

For patients with AIS due to LVO who require early EVT, any improvement in the workflow time or transport efficiency that expedites their arrival at a CSC could potentially shorten their time to treatment. However, the arrival pathways may vary between geographical regions and countries, depending on local EMT circumstances, including staff availability, access to triage on the spot, and the proximity and availability of nearby primary and comprehensive stroke centers. Previous studies report that about 50% of EVT patients were treated after interhospital transfer, (13, 14) a much higher fraction than the 20.8% observed in our patient cohort. These differences likely result from the high population density in cities in Taiwan. No consensus has been reached on the effect of transport method on post-EVT outcomes, particularly in the Asia-Pacific region. Several studies suggest that transport mode was important to reduce delays in treatment but did not significantly affect post-EVT outcomes (14–17). For example, a study using a nationwide stroke registry in Singapore found that patients transported by emergency medical services (EMS) were more likely to receive timely stroke evaluation and treatment (door-to-needle time ≤ 60 min) than self-transported patients (15). Similarly, a study conducted in Beijing, China, found that transport mode did not result in significant differences in post-EVT outcomes despite the fact that the onset-to-needle time was longer for direct admission to a CSC compared to transfer from PSC to CSC (16). In South Korea, establishing a fast-tracked interhospital transfer system was suggested to improve IVT initiation in the drip-and-ship (at PSC) approach compared to direct CSC admission (17). Outside of the East Asian countries, a cohort of EVT-treated patients in central Israel was observed to have comparable OTP times and clinical outcomes between direct and transfer groups (18). These studies suggest that not only workflow time but also geographical regions could affect post-EVT outcomes.

Our study found that the arrival pathway was significantly associated with post-EVT 3-month functional independence and that the optimal arrival pathway differed depending on whether or not the time to treatment was delayed. To administer treatment during the early time window, direct admission is recommended for timely EVT. Studies from Japan and Australia have reported similar findings, where interhospital transfer worsened the odds of achieving a 90-day mRS of 0–2 and increased the OTP time compared to direct admission for patients receiving EVT for AIS (19, 20). An analysis of South Korean national stroke audit data also found that direct dispatch to a CSC resulted in better EVT rates and functional outcomes compared to an initial dispatch to a PSC (21).

Studies outside of the Asia-Pacific region have also shown mixed results. Some reported comparable 3-month post-EVT outcomes between direct admission and transfer route for patients with anterior circulation LVO (22) and basilar artery occlusion (23). Studies supportive of direct admission to a CSC for timely treatment reported that secondary transfers from PSCs were associated with worse functional independence in Spain (24). In a cohort of 650 AIS-LVO patients from the international COMPLETE registry, the investigators observed that transferred patients were treated with EVT later and were less likely to achieve 90-day functional independence than were those transported directly to CSCs (25). A regional stroke network in rural Germany also reported that the transfer approach was inferior to the direct transport to CSC in terms of 3-month post-EVT functional outcomes (26). Recent randomized clinical trials, including RACECAT, reported comparable outcomes between direct-to-CSC and transfer approaches (7, 8). The benefit of saving time by direct transport was counterbalanced by worse functional outcomes in ICH patients (27). Nevertheless, other studies suggest that geographical and healthcare structural differences likely influenced post-EVT functional outcomes. For example, for patients with LVO-induced stroke from the RACECAT trial conducted in nonurban Catalonia, Spain, were less likely to receive EVT and had worse mRS scores than did propensity-matched patients living in urban areas of the same region (28). The authors further showed that each 30-min delay in time-to-EMS evaluation was associated with a 1.03-folds increased risk of 90-day disability in patients with severe stroke who were taken to a PSC first (29). Similarly, being transported to a PSC first was associated with worse disability outcomes in patients evaluated by EMS >2 h after symptom onset (29). However, transport time savings for thrombolysis using the PSC-first approach varied across different networks (27). Compared to AIS patients in less populated regions, those in more populated regions received thrombolysis faster but EVT within a similar timeframe, potentially resulting in more comparable outcomes between the two transport strategies in less populated areas (27). These findings raise concerns about the current EMS triage capabilities and the availability of non-imaging evaluation tools for different stroke types, highlighting the need for further investigations into the pros and cons of transporting patients with suspected AIS directly to a CSCs or via PSC (9, 10).

We found an interaction between OTP time and arrival pathway associating with the 3-month functional independence outcome. For patients who arrived at the CSC directly, 3-month functional independence was most closely associated with time to treatment. In contrast, in the transfer group, the probability of achieving 3-month functional independence was less associated with OTP time. This finding could result from differences in EVT feasibility between hospitals and the medical assistance provided by the PSC. EVT of the anterior circulation beyond 8 h after onset is not covered by the national health insurance of Taiwan. Additionally, tissue-clock evaluation to justify the use of any intervention was widely used in the later treatment window. We observed that direct admission to a CSC was recommended during the early treatment window (< 6 h) and for patients with higher GCS identified by EMT for timely management. In the later time window (6–24 h), arrival by transfer was associated with a higher likelihood of 3-month functional independence for younger patients and those with a lower NIHSS and higher ASPECTS score before EVT. The cost-effectiveness regarding time and distance required to transport patients to a PSC and CSC within 8 h of symptom onset was evaluated in a US study (30). The authors showed that the direct-to-CSC pathway was the most cost-effective approach when the distances to reach a PSC and a CSC were similar. In contrast, the transfer pathway was the preferred strategy when the PSC and CSC were both far from the location of onset (>1.5 h away) (30). While the distance between the PSC and CSC was not examined in our study, Taiwan's healthcare infrastructure is geographically similar to large urban settings in which the tertiary care, stroke center, and EMS are easily accessible within 1.5 h. This factor likely explains our finding that direct arrival to a CSC was associated with more favorable outcomes compared to transfer from a PSC during the early treatment window.

In our cohort, a higher prevalence of carotid artery occlusions was observed in the transfer group, a factor that could theoretically contribute to lower baseline ASPECTS and poorer clinical outcomes. To address this potential confounding, we performed additional statistical validations. First, the Variance Inflation Factor (VIF) for carotid artery occlusion and ASPECTS was 1.01, indicating negligible multicollinearity and confirming that these variables act as independent predictors in our model. Furthermore, within the transfer group, baseline ASPECTS did not differ significantly between patients with and without carotid artery occlusion (median 7 [IQR 6–9] vs. 8 [IQR 6–9], *p* = 0.084). This suggests that carotid artery occlusion was not the primary driver of lower baseline ASPECTS in transferred patients. Most importantly, in multivariable analysis that included both ASPECTS and carotid artery occlusion and were stratified by onset-to-puncture interval, the transfer pathway remains an independent predictor of 3-month functional independence (mRS 0–2) (Table 3). Therefore, carotid artery occlusion and ASPECTS do not fully explain the pathway–outcome association, although residual confounding cannot be excluded; further studies and sensitivity analyses (models with and without ASPECTS, and stratified analyses by carotid status) are warrant to confirm the robustness of these findings.

Although mobile stroke units (MSUs) are not currently available in Taiwan, their potential importance for prehospital stroke care warrants acknowledgment. MSUs represent a transformative advancement in prehospital care by addressing the critical “time is brain” principle through the delivery of rapid diagnosis and treatment directly at the point of patient contact (31). Recent clinical evidence, specifically from the Berlin prehospital Or usual delivery of acute stroke care (B_PROUD) and Benefits of Stroke Treatment Delivered by a mobile stroke unit Compared with standard management by emergency medical services (BEST-MSU) trials, demonstrated that MSUs—ambulances equipped with CT scanners, point-of-care laboratories, and specialized teams—significantly improved functional outcomes and reduced treatment delays (32, 33). A recent systematic review, including five randomized controlled trials, six observational studies, and two meta-analyses, reported that MSUs reduced the median onset-to-needle time for thrombolysis by 20–41 min compared to standard EMS. This time efficiency substantially increased golden-hour thrombolysis rates (from < 5% with EMS to 21%−33% with MSUs) and was associated with improved 90-day functional outcomes (mRS 0–1) in patients with AIS (34). While resource constraints currently preclude the implementation of MSUs in Taiwan and may limit prehospital optimization, their proven benefits underscore the importance of considering such innovations in future stroke system planning.

While this study used a relatively large, prospectively enrolled registry that reflected real-world scenarios, several limitations should be acknowledged. TREAT-AIS only enrolled patients who received EVT and not all stroke patients with suspected LVO. Additionally, due to the nature of TREAT-AIS, this study focused on an East Asian population with a high population density. Because PSC arrival time is not a mandatory registry field, only 99 patients had documented PSC data. The discrepancy in sample size between the direct and transfer groups likely reflects regional geographical characteristics rather than prehospital LVO screening, which has not yet been adopted nationwide. The ratio of direct-to-CSC admissions is known to fluctuate significantly based on population density and the proximity of thrombectomy centers. For instance, while the MR CLEAN registry (Netherlands)—a country with land area, population size and the number of thrombectomy centers comparable to Taiwan—reported a lower proportion of direct admissions (45%−55%), (35) the East Asian metropolitan K-NET registry reported a direct-to-CSC rate as high as 92% (36). Our cohort's distribution (79% direct-to-CSC) sits between these international benchmarks, suggesting that differences in healthcare infrastructure and regional hospital distribution may explain the intermediate ratio. While the registry was initiated in 2019, the primary impact of the COVID-19 pandemic on stroke workflows in Taiwan was delayed until the major outbreaks of 2021. Analysis of Supplementary Tables S5, S6 demonstrates that the pandemic did not significantly affect workflow and post-EVT outcomes for either direct-to-CSC or transfer patients. Nevertheless, the potential influence of the pandemic on stroke care during certain periods is acknowledged as a limitation. Taken together, these factors could limit the generalizability of the reported findings. The nonrandomized observational study design precludes causal inference. The distances between the location of onset, PSC, and CSC were not reported in the registry, hindering further geographical investigations into route optimization to avoid workflow or treatment delays. TREAT-AIS does not explicitly record which image source was used to derive ASPECTS in transferred patients. Consequently, observed differences in baseline ASPECTS between the transfer and direct groups may reflect selection/referral practices, timing-related infarct progression, or heterogeneity in CT acquisition and reader interpretation across centers, any of which could introduce bias. Furthermore, no mismatch profile was recorded for the later treatment window, which has been identified as a promising proxy for outcomes.

## Conclusions

For patients receiving EVT for AIS, direct transport to a CSC was associated with greater 3-month functional independence than was transfer from a PSC. During the early treatment window, proactive EVT treatment is recommended regardless of the arrival pathway, especially for patients with higher GCS scores. However, patients who arrive via transfer route may still benefit substantially from EVT during the later treatment window, especially those who are younger, use antiplatelet agents, and have lower NIHSS and higher ASPECTS scores. A randomized study is warranted to examine the predictors of favorable post-EVT outcomes within a 24-h window to facilitate timely treatment.

## Data Availability

The raw data supporting the conclusions of this article will be made available by the authors, without undue reservation.
