# Supplementary material for: Influence of interhospital transfer on endovascular thrombectomy outcome in acute ischemic stroke patients: an analysis of the TREAT-AIS registry
Source: Front Neurol. 2026 Mar 16;17:1743928. doi: 10.3389/fneur.2026.1743928 (PMC13033569; doi:10.3389/fneur.2026.1743928)
Supplement: Supplementary file 1 [file Table_1.pdf]

**Supplementary Table S1. Comparison of EVT outcomes by time of presentation (daytime vs. nighttime) in patients who went directly to CSC**

| Outcome | Day (08:00–17:00) | Night | P value |
|---|---|---|---|
| Total Cases | N = 851 | N = 582 | |
| Functional outcomes, n (%) | | | |
| Final TICI (mTICI 2b-3) | 718 (84.4) | 470 (80.8) | 0.086 |
| Symptomatic ICH | 36 (4.2) | 26 (4.5) | 0.895 |
| 3-month mRS 0-2 (n=1315) | 263 (33.5) | 164 (30.9) | 0.337 |
| 3-month mRS mortality | 151 (17.4) | 88 (15.1) | 0.195 |
| Workflow outcomes, median (IQR)(min) | | | |
| Onset-to-door (n=1136) | 72 (38–169) | 75 (41–171) | 0.494 |
| Door-to-puncture (n=1262) | 150 (111–196) | 151 (125–189) | 0.111 |
| Onset-to-puncture (n=1255) | 225 (170–345) | 236.5 (181.5–344.5) | **0.049** |
| Onset to puncture <6 hr, n (%) (n=1255) | 383 (76.6) | 583 (77.2) | 0.837 |

P <0.05 shown in bold.

CSC, comprehensive stroke center; door, arrival at CSC; EVT, endovascular thrombectomy; ICH, intracranial hemorrhage; IQR, interquartile range; mRS, modified Rankin Scale; mTICI, modified Thrombolysis in Cerebral Infarction; Onset, symptom onset; puncture, EVT initiation; TICI, Thrombolysis in Cerebral Infarction.

**Supplementary Table S2. Comparison of EVT outcomes by time of presentation (daytime vs. nighttime) in patients transferred from a PSC**

| Outcome | Day (08:00–17:00) | Night | P value |
|---|---|---|---|
| Total Cases | N = 201 | N = 179 | |
| Functional outcomes, n (%) | | | |
| Final TICI (mTICI 2b-3) | 174 (86.6) | 155 (86.6) | 1.000 |
| Symptomatic ICH | 12 (5.9) | 18 (10.1) | 0.182 |
| 3-month mRS 0-2 (n=335) | 42 (23.5) | 39 (25.0) | 0.799 |
| 3-month mRS mortality | 39 (19.4) | 32 (17.9) | 0.792 |
| Workflow outcomes, median (IQR)(min) | | | |
| Onset-to-door (n=341) | 236 (175–327) | 236 (182–312) | 0.948 |
| Door-to-puncture (n=364) | 103 (75–136) | 103.5 (71–128) | 0.953 |
| Onset-to-puncture (n=341) | 330.5 (269–442) | 340 (283–417) | 0.754 |
| Onset to puncture <6 hr, n (%) (n=341) | 105 (58.9) | 93 (57.1) | 0.743 |

Door, arrival at CSC; EVT, endovascular thrombectomy; ICH, intracranial hemorrhage; IQR, interquartile range; mRS, modified Rankin Scale; mTICI, modified Thrombolysis in Cerebral Infarction; Onset, symptom onset; PSC, primary stroke center; puncture, EVT initiation; TICI, Thrombolysis in Cerebral Infarction.

**Supplementary Table S3. Comparison of EVT outcomes by time of presentation (weekday vs. weekend) in patients direct to CSC**

| Outcome | Weekday | Weekend | P value |
|---|---|---|---|
| Total Cases | N = 1055 | N = 378 | |
| Functional outcomes, n (%) | | | |
| Final TICI (mTICI 2b-3) | 880 (83.4) | 307 (81.2) | 0.341 |
| Symptomatic ICH | 44 (4.2) | 18 (4.8) | 0.659 |
| 3-month mRS 0-2 (n=1315) | 320 (33.0) | 107 (30.9) | 0.504 |
| 3-month mRS mortality | 190 (18.0) | 49 (12.9) | **0.024** |
| Workflow outcomes, median (IQR)(min) | | | |
| Onset-to-door (n=1136) | 73 (40–170) | 73 (40–178) | 0.767 |
| Door-to-puncture (n=1262) | 147 (113–192) | 158 (126–197) | **0.007** |
| Onset-to-puncture (n=1254) | 228 (173–349) | 232 (180–335) | 0.493 |
| Onset to puncture <6 hr, n (%) (n=1254) | 717 (77.0) | 248 (76.8) | 0.939 |

P <0.05 shown in bold.

CSC, comprehensive stroke center; door, arrival at CSC; EVT, endovascular thrombectomy; ICH, intracranial hemorrhage; IQR, interquartile range; mRS, modified Rankin Scale; mTICI, modified Thrombolysis in Cerebral Infarction; Onset, symptom onset; puncture, EVT initiation; TICI, Thrombolysis in Cerebral Infarction.

**Supplementary Table S4. Comparison of EVT outcomes by time of presentation (weekday vs. weekend) in patients transferred from a PSC**

| Outcome | Weekday | Weekend | P value |
|---|---|---|---|
| Total Cases | N = 289 | N = 92 | |
| Functional outcomes, n (%) | | | |
| Final TICI (mTICI 2b-3) | 251 (86.9) | 79 (85.9) | 0.861 |
| Symptomatic ICH | 24 (8.3) | 6 (6.5) | 0.663 |
| 3-month mRS 0-2 (n=336) | 62 (24.0) | 20 (25.6) | 0.765 |
| 3-month mRS mortality | 55 (19.0) | 16 (17.4) | 0.878 |
| Workflow outcomes, median (IQR)(min) | | | |
| Onset-to-door (n=341) | 240 (185–333) | 224.5 (152–285) | **0.049** |
| Door-to-puncture (n=364) | 98.5 (72–128) | 111.5 (83.5–146) | **0.039** |
| Onset-to-puncture (n=342) | 343 (271–435) | 321 (275–412) | 0.397 |
| Onset to puncture <6 hr*, n (%) (n=342) | 144 (56.0) | 54 (63.5) | 0.255 |

P <0.05 shown in bold.

Door, arrival at CSC; EVT, endovascular thrombectomy; ICH, intracranial hemorrhage; IQR, interquartile range; mRS, modified Rankin Scale; mTICI, modified Thrombolysis in Cerebral Infarction; Onset, symptom onset; PSC, primary stroke center; puncture, EVT initiation; TICI, Thrombolysis in Cerebral Infarction.

**Supplementary Table S5. Comparison of EVT outcomes by presentation period (non-COVID vs. COVID) in patients direct to CSC**

| Outcome | Non-COVID (2019–2020) | COVID (2021–2022) | P value |
|---|---|---|---|
| Total Cases | N = 580 | N = 853 | |
| Functional outcomes, n (%) | | | |
| Final TICI (mTICI 2b-3) | 487 (83.9) | 700 (82.1) | 0.355 |
| Symptomatic ICH | 26 (4.5) | 36 (4.2) | 0.895 |
| 3-month mRS 0-2 (n=1315) | 192 (34.4) | 235 (31.0) | 0.211 |
| 3-month mRS mortality | 88 (15.1) | 151 (17.7) | 0.220 |
| Workflow outcomes, median (IQR)(min) | | | |
| Onset-to-door (n=1136) | 68 (38–183) | 75 (41–164) | 0.380 |
| Door-to-puncture (n=1262) | 151 (111–192) | 151 (121–194) | 0.214 |
| Onset-to-puncture (n=1254) | 226 (163–350) | 230 (180–341) | 0.176 |
| Onset to puncture <6 hr, n (%) (n=1254) | 388 (76.2) | 577 (77.5) | 0.633 |

CSC, comprehensive stroke center; door, arrival at CSC; EVT, endovascular thrombectomy; ICH, intracranial hemorrhage; IQR, interquartile range; mRS, modified Rankin Scale; mTICI, modified Thrombolysis in Cerebral Infarction; Onset, symptom onset; puncture, EVT initiation; TICI, Thrombolysis in Cerebral Infarction.

**Supplementary Table S6. Comparison of EVT outcomes by presentation period (non-COVID vs. COVID) in patients transferred from a PSC**

| Outcome | Non-COVID (2019–2020) | COVID (2021–2022) | P value |
|---|---|---|---|
| Total Cases | N = 149 | N = 232 | |
| Functional outcomes, n (%) | | | |
| Final TICI (mTICI 2b-3) | 128 (85.9) | 202 (87.1) | 0.759 |
| Symptomatic ICH | 9 (6.0) | 21 (9.1) | 0.334 |
| 3-month mRS 0-2 (n=336) | 36 (25.7) | 46 (23.5) | 0.699 |
| 3-month mRS mortality | 33 (22.2) | 38 (16.4) | 0.178 |
| Workflow outcomes, median (IQR)(min) | | | |
| Onset-to-door (n=341) | 251 (186–341) | 231.5 (170–309) | 0.167 |
| Door-to-puncture (n=374) | 101 (66–129) | 104 (80–132) | 0.151 |
| Onset-to-puncture (n=342) | 349 (275–430) | 325 (274–426) | 0.573 |
| Onset to puncture <6 hr, n (%) (n=342) | 74 (55.6) | 124 (59.3) | 0.503 |

P <0.05 shown in bold.

Door, arrival at CSC; EVT, endovascular thrombectomy; ICH, intracranial hemorrhage; IQR, interquartile range; mRS, modified Rankin Scale; mTICI, modified Thrombolysis in Cerebral Infarction; Onset, symptom onset; PSC, primary stroke center; puncture, EVT initiation; TICI, Thrombolysis in Cerebral Infarction.

**Supplementary Table S7.** Factors associated with 3-month mRS 0–2 by logistic regression analysis

| Variable | Univariable | | Multivariate | |
|---|---|---|---|---|
| | OR (95% CI) | P | aOR (95% CI) | P |
| Method of arrival (Ref: Direct CSC) | | | | |
| Transferred from PSC | 0.67 (0.51–0.88) | **0.004** | 0.76 (0.55–1.07) | 0.112 |
| Age | 0.96 (0.95–0.97) | **<0.001** | 0.96 (0.95–0.97) | **<0.001** |
| Hypertension | 0.68 (0.54–0.86) | **0.001** | 0.85 (0.64–1.14) | 0.273 |
| Previous transient ischemic attack | 1.32 (0.68–2.58) | 0.413 | – | - |
| Dyslipidemia | 1.19 (0.96–1.46) | 0.106 | – | - |
| Cancer | 0.63 (0.45–0.88) | **0.006** | 0.50 (0.33–0.74) | **<0.001** |
| Atrial fibrillation | 0.72 (0.59–0.89) | **0.002** | 1.15 (0.88–1.50) | 0.306 |
| Antiplatelet medication | 0.97 (0.74–1.26) | 0.797 | – | - |
| Lipid-lowering drug | 1.16 (0.89–1.51) | 0.287 | – | - |
| NIHSS at baseline | 0.92 (0.90–0.93) | **<0.001** | 0.94 (0.92–0.96) | **<0.001** |
| Intravenous thrombolysis | 1.81 (1.46–2.24) | **<0.001** | 1.40 (1.07–1.82) | **0.014** |
| ASPECTS before EVT | 1.21 (1.14–1.29) | **<0.001** | 1.14 (1.06–1.23) | **<0.001** |
| Carotid artery involvement (Ref: No) | 0.74 (0.58–0.94) | **0.016** | 0.79 (0.59–1.06) | 0.122 |
| M2-3 artery involvement (Ref: others) | 1.26 (0.98–1.62) | 0.066 | – | - |
| Onset-to-puncture >6 h (Ref: <6 h) | 0.69 (0.53–0.89) | **0.004** | 0.75 (0.55–1.03) | 0.079 |
| GCS score | 1.18 (1.14–1.23) | **<0.001** | 1.07 (1.02–1.12) | **0.005** |

aOR, adjusted odds ratio; ASPECTS, Alberta Stroke Program Early Computed Tomography Score; CI, confidence interval; CSC, comprehensive stroke center; EVT, endovascular thrombectomy; GCS, Glasgow Coma Scale; mRS, modified Rankin Scale; NIHSS, National Institute of Health Stroke Scale; PSC, primary stroke center; OR, odds ratio.

Multivariate model: variables with P < 0.05 in the univariate analysis were included in the multivariate model.

**Supplementary Table S8. Descriptive distributions and univariate analyses for factors associated with sICH, stratified by onset-to-puncture interval**

| | Onset to puncture time < 6 hours | | | | | | Onset to puncture time >= 6 hours | | | | | |
|---|---|---|---|---|---|---|---|---|---|---|---|---|
| | sICH | | Non-sICH | | | | sICH | | Non-sICH | | | |
| Variable | N=60 | % | N=1108 | % | OR (95%CI) | P value | N=17 | % | N=420 | % | OR (95%CI) | P value |
| Method of arrival (Ref:Direct CSC) | | | | | | | | | | | | |
| Transferred from PSC | 17 | 28.3 | 182 | 16.4 | 2.02 (1.13–3.63) | 0.018 | 8 | 47.1 | 136 | 32.3 | 1.86 (0.70–4.93) | 0.211 |
| Age, median (IQR) | 73 | 63.5–81 | 72 | 64–81 | 1.00 (0.98–1.02) | 0.770 | 72 | 62–81 | 73 | 62–82 | 1.01 (0.97–1.04) | 0.727 |
| Hypertension | 49 | 81.7 | 810 | 72.8 | 1.67 (0.86–3.25) | 0.134 | 13 | 76.5 | 300 | 71.3 | 1.31 (0.42–4.10) | 0.642 |
| Previous transient ischemic attack | 0 | 0.0 | 24 | 2.2 | – | – | 0 | 0.0 | 14 | 3.3 | – | – |
| Dyslipidemia | 25 | 41.7 | 581 | 52.2 | 0.65 (0.39–1.11) | 0.114 | 7 | 41.2 | 228 | 54.2 | 0.59 (0.22–1.59) | 0.298 |
| Cancer | 2 | 3.3 | 152 | 13.7 | 0.22 (0.05–0.90) | 0.036 | 4 | 23.5 | 57 | 13.5 | 1.97 (0.62–6.24) | 0.252 |
| Atrial fibrillation | 35 | 58.3 | 627 | 56.3 | 1.09 (0.64–1.84) | 0.761 | 8 | 47.1 | 182 | 43.2 | 1.17 (0.44–3.08) | 0.755 |
| Antiplatelet medication | 13 | 21.7 | 219 | 19.7 | 1.13 (0.60 – 2.12) | 0.706 | 4 | 23.5 | 71 | 16.9 | 1.52 (0.59–4.79) | 0.477 |
| Lipid-lowering drug | 15 | 25.0 | 205 | 18.4 | 1.48 (0.81 – 2.70) | 0.206 | 2 | 11.8 | 65 | 15.4 | 0.73 (0.16–3.27) | 0.681 |
| NIHSS at baseline, median (IQR) | 19.5 | 16–23 | 18 | 13–24 | 1.02 (0.98–1.06) | 0.269 | 19 | 13–23 | 16 | 11–22 | 1.04 (0.97–1.11) | 0.281 |
| Intravenous thrombolysis | 30 | 52.6 | 526 | 49.8 | 1.12 (0.66–1.91) | 0.678 | 1 | 5.9 | 59 | 14.8 | 0.36 (0.05–2.76) | 0.325 |
| ASPECTS before EVT, median (IQR) | 7.5 | 6–9 | 9 | 7–10 | 0.82 (0.73–0.92) | <0.001 | 8 | 7–10 | 8 | 7–9 | 0.97 (0.76–1.23) | 0.773 |
| Carotid artery involvement (Ref: No) | 20 | 33.3 | 290 | 26.1 | 1.42 (0.82–2.47) | 0.215 | 8 | 47.1 | 92 | 21.9 | 3.18 (1.19–8.47) | 0.021 |
| M2-3 artery involvement (Ref: others) | 12 | 20.0 | 249 | 22.4 | 0.87 (0.45–1.66) | 0.667 | 0 | 0.0 | 91 | 21.6 | – | – |
| GCS score, median (IQR) | 11 | 9–15 | 11 | 9–15 | 0.98 (0.91–1.07) | 0.695 | 11 | 9–15 | 11 | 9–15 | 1.00 (0.88–1.13) | 0.994 |

ASPECTS, Alberta Stroke Program Early Computed Tomography Score; CI, confidence interval; CSC, comprehensive stroke center; EVT, endovascular thrombectomy; GCS, Glasgow Coma Scale; IQR, interquartile range; NIHSS, National Institute of Health Stroke Scale; PSC, primary stroke center; OR, odds ratio; sICH, symptomatic intracranial hemorrhage.

**Supplementary Table S9. Descriptive distributions and univariate analyses for factors associated with mRS, stratified by onset-to-puncture interval**

| | Onset to puncture time < 6 hours | | | | | | Onset to puncture time >= 6 hours | | | | | |
|---|---|---|---|---|---|---|---|---|---|---|---|---|
| | 3-month mRS 0-2 | | 3-month mRS >2 | | | | 3-month mRS 0-2 | | 3-month mRS >2 | | | |
| Variable | N=354 | % | N=701 | % | OR (95%CI) | P value | N=105 | % | N=303 | % | OR (95%CI) | P value |
| Method of arrival (Ref:Direct CSC) | | | | | | | | | | | | |
| Transferred from PSC | 39 | 10.9 | 136 | 19.4 | 0.51 (0.35–0.75) | <0.001 | 37 | 34.9 | 93 | 30.7 | 1.21 (0.76–1.93) | 0.423 |
| Age, median (IQR) | 68 | 60–75 | 76 | 65–83 | 0.96 (0.95–0.97) | <0.001 | 65 | 57–74 | 77 | 65–84 | 0.95 (0.94–0.97) | <0.001 |
| Hypertension | 249 | 69.6 | 528 | 75.2 | 0.75 (0.57–0.99) | 0.049 | 67 | 63.2 | 230 | 75.9 | 0.55 (0.34–0.88) | 0.012 |
| Previous transient ischemic attack | 11 | 3.1 | 8 | 1.1 | 2.75 (1.10–6.89) | 0.031 | 3 | 2.8 | 11 | 3.6 | 0.77 (0.21–2.83) | 0.697 |
| Dyslipidemia | 199 | 55.6 | 349 | 49.7 | 1.27 (0.98–1.64) | 0.071 | 60 | 56.6 | 159 | 52.5 | 1.18 (0.76–1.84) | 0.463 |
| Cancer | 33 | 9.2 | 109 | 15.5 | 0.55 (0.37–0.83) | 0.005 | 11 | 10.4 | 47 | 15.5 | 0.63 (0.31–1.27) | 0.196 |
| Atrial fibrillation | 184 | 51.4 | 415 | 59.1 | 0.73 (0.57–0.95) | 0.017 | 38 | 35.8 | 138 | 45.5 | 0.67 (0.42–1.06) | 0.084 |
| Antiplatelet medication | 76 | 21.2 | 138 | 19.7 | 1.10 (0.80–1.51) | 0.547 | 13 | 12.3 | 58 | 19.1 | 0.59 (0.31–1.13) | 0.111 |
| Lipid-lowering drug | 75 | 20.9 | 127 | 18.1 | 1.20 (0.87–1.65) | 0.263 | 19 | 17.9 | 43 | 14.2 | 1.32 (0.73–2.39) | 0.357 |
| NIHSS at baseline, median (IQR) | 15 | 11–21 | 20 | 15–25 | 0.91 (0.90–0.93) | <0.001 | 11.5 | 8–16 | 18 | 13–24 | 0.90 (0.86–0.93) | <0.001 |
| Intravenous thrombolysis | 205 | 58.6 | 295 | 44.4 | 1.77 (1.36–2.30) | <0.001 | 17 | 16.7 | 38 | 13.3 | 1.30 (0.69–2.42) | 0.409 |
| ASPECTS before EVT, median (IQR) | 9 | 8–10 | 8 | 7–10 | 1.16 (1.07–1.25) | <0.001 | 9 | 7–10 | 8 | 6–9 | 1.27 (1.11–1.46) | <0.001 |
| Carotid artery involvement (Ref: No) | 84 | 23.5 | 198 | 28.2 | 0.78 (0.58–1.05) | 0.099 | 18 | 17.0 | 74 | 24.4 | 0.63 (0.36–1.12) | 0.117 |
| M2-3 artery involvement (Ref: others) | 86 | 24.0 | 147 | 20.9 | 1.19 (0.88–1.62) | 0.252 | 26 | 24.5 | 59 | 19.5 | 1.34 (0.79–2.27) | 0.270 |
| GCS score, median (IQR) | 13.5 | 10–15 | 11 | 9–15 | 1.19 (1.14–1.24) | <0.001 | 15 | 10–15 | 11 | 8–15 | 1.16 (1.09–1.24) | <0.001 |

ASPECTS, Alberta Stroke Program Early Computed Tomography Score; CI, confidence interval; CSC, comprehensive stroke center; EVT, endovascular thrombectomy; GCS, Glasgow Coma Scale; IQR, interquartile range; mRS, modified Rankin Scale; NIHSS, National Institute of Health Stroke Scale; PSC, primary stroke center; OR, odds ratio.
